# The Relationship Between Postoperative Outcomes of Gynecologic Patients After Receiving the Enhanced Recovery After Surgery (ERAS) Protocol Versus Narcotic Medication for Pain Management

**DOI:** 10.7759/cureus.81420

**Published:** 2025-03-29

**Authors:** Ovgu Barut, Dynora Pierre-Louis, Jose Luis Terrazas, Adi Abramovici

**Affiliations:** 1 Obstetrics and Gynecology, HCA Healthcare, Margate, USA; 2 Obstetrics and Gynecology, HCA Florida Northwest Hospital, Margate, USA; 3 Maternal-Fetal Medicine, Sinai Perinatal, Plantation, USA

**Keywords:** eras protocol, gynecologic surgery, gynecology, minimally invasive surgery, postoperative pain

## Abstract

This retrospective research project will assess the utilization of the Enhanced Recovery After Surgery (ERAS) protocol compared to narcotic treatment in the postoperative course of benign gynecological surgeries. We intend to study the potential relationship between the frequency of readmission rates, deep vein thrombosis (DVT), pulmonary embolism (PE), length of stay, and opioid use in the pre-discharge period in those who receive the ERAS protocol versus narcotics for pain management. The goal is also to increase the implementation of the ERAS protocol in our hospital if it is shown to be superior in this project. We hypothesize that the rate of readmission, frequency of DVT, PE, length of stay, and opioid use in the pre-discharge period will be lower in patients receiving the ERAS protocol. Female patients older than 18 years old who underwent robotic/laparoscopic/abdominal benign gynecologic surgeries in the inpatient setting between 2020 and 2023 in the HCA Florida East Division hospitals were included in this study. The analysis indicates that being in the narcotics group (incidence rate ratio (IRR) = 1.242, p = 0.001) or the ERAS + narcotics group (IRR = 1.886, p < 0.001) is associated with a significantly longer length of stay compared to the ERAS group. A grouped Charlson Index score of 1 (IRR = 1.285, p < 0.001) or 2 or higher (IRR = 2.000, p < 0.001) is also associated with a longer length of stay. Other covariates, including age, race, BMI, and smoking status, did not show statistically significant associations. The results show that being in the ERAS + narcotics group is significantly associated with increased odds of readmission (OR = 3.507, p < 0.001) compared to the ERAS group (readmission is analyzed regardless of specific diagnosis). Older age groups, specifically 45-64 years (OR = 0.574, p = 0.001) and 65 years and over (OR = 0.439, p < 0.001), are associated with lower odds of readmission compared to the 18-44 years group. Older patients may receive more comprehensive care, discharge planning, medications, and follow-ups tailored to their profile, hence returning less compared to the younger group. A grouped Charlson Index score of 1 (OR = 1.692, p = 0.019) or 2 or higher (OR = 3.086, p < 0.001) is significantly associated with increased odds of readmission. We conclude that the utilization of the ERAS protocol compared to narcotic treatment in the postoperative course of benign gynecological surgeries is superior to narcotic treatment and narcotic treatment combined with the ERAS protocol. The ERAS group was associated with shorter length of stay and decreased rates of readmission. Implementing the ERAS protocol as a standard of care is an important step shown to decrease hospital costs, improve patient outcomes, and improve hospital quality.

## Introduction

Originally developed by the field of colorectal surgery, the Enhanced Recovery After Surgery (ERAS) protocol is defined as a multimodal approach directed at optimizing the experience of the patient, including standardized perioperative care, and improving outcomes of surgery [1]. The primary objective is to maintain normal physiology in patients undergoing surgery [2].

The ERAS model was then modified for gynecologic patients. The gynecologic ERAS protocol emphasizes early ambulation and involves increased focus on multimodal pain management. As part of the postoperative pain management, the protocol uses regular scheduled doses of nonsteroidal anti-inflammatory drugs (NSAIDs) and acetaminophen to spare narcotic use and decrease the use of opioid consumption and associated adverse effects [3].

Readmission rates are defined as the percentage of admitted patients who return to the hospital within 30 days of discharge. It has been defined as a measure of hospital quality and effective care. Analyzing readmission rates, length of stay, and patient satisfaction may provide insight into patient care and hospital quality. Research suggests 5.9% of patients develop dependence on opioids related to prescription opioids [4]. Much of the data already known about ERAS protocol vs. narcotics arises from gynecologic oncology and colorectal surgery, but data from benign gynecology cases are understudied.

This project will assess the utilization of the ERAS protocol compared to narcotic treatment in the postoperative course of benign gynecological surgeries and, therefore, is intended to promote decreased use of opioid narcotics and increase the implementation of the ERAS protocol. We intend to study the potential relationship between the frequency of readmission rates, deep vein thrombosis (DVT), pulmonary embolism (PE), length of stay, and opioid use in the pre-discharge period in those who receive the ERAS protocol versus narcotics for pain management. The goal is also to increase the implementation of the ERAS protocol in our hospital if it is shown to be superior in this project. Due to the ERAS protocol’s emphasis on early ambulation, limited narcotic use, and minimally invasive surgery, we hypothesize that the rate of readmission, frequency of DVT, PE, length of stay, and opioid use in the pre-discharge period will be lower in patients receiving the ERAS protocol.

## Materials and methods

Secondary analysis of de-identified existing data was performed. The HCA corporate database was queried. The regression analysis examined the relationship between race and the frequency and severity of allergic reactions, while controlling for age. The variables on which data were collected include age, race, obesity, parity, smoking status, diabetes mellitus (DM), chronic hypertension (CHTN), and readmission rates. The predictor variables include the ERAS protocol (opioid-naïve order set on the electronic medical record (EMR) system) vs. narcotics. Control variables include age, race, obesity, parity, smoking status, DM, and CHTN. Outcome variables include rate of readmission rates, frequency of DVT, PE, length of stay, and opioid use in the pre-discharge period. Inclusion criteria include those treated with the ERAS protocol and narcotics after robotic/laparoscopic/abdominal benign gynecologic surgeries. Exclusion criteria include those who received pain management protocol other than the ERAS protocol and narcotics in the immediate postoperative period after gynecologic surgery, those without chronic narcotic use, and those with unscheduled surgery.

The total population size for this project was 3,919 people, with 1,504 people enrolled in the narcotic group, 81 people enrolled in the ERAS + narcotic group, and 304 people enrolled in the ERAS group. Regression analysis was used to compare the narcotics group with the ERAS + narcotics group. Logistic regression analyzed factors associated with 30-day readmission. Negative binomial regression was performed to analyze the length of stay.

## Results

The analysis indicates that being in the narcotics group (incidence rate ratio (IRR) = 1.242, p = 0.001) or the ERAS + narcotics group (IRR = 1.886, p < 0.001) is associated with a significantly longer length of stay compared to the ERAS group. A grouped Charlson Index score of 1 (IRR = 1.285, p < 0.001) or 2 or higher (IRR = 2.000, p < 0.001) is also associated with a longer length of stay. Other covariates, including age, race, BMI, and smoking status, did not show statistically significant associations. The results show that being in the ERAS + narcotics group is associated with increased odds of readmission (OR = 3.507, p < 0.001) compared to the ERAS group (readmission is analyzed regardless of specific diagnosis). Older age groups, specifically 45-64 years (OR = 0.574, p = 0.001) and 65 years and over (OR = 0.439, p < 0.001), are associated with lower odds of readmission compared to the 18-44 years group. Older patients may receive more comprehensive care, discharge planning, medications, and follow-ups tailored to their profile, hence returning less compared to the younger group. A grouped Charlson Index score of 1 (OR = 1.692, p = 0.019) or 2 or higher (OR = 3.086, p < 0.001) is significantly associated with increased odds of readmission. The total population size for this project was 3,919 people, with 1,504 people enrolled in the narcotic group, 81 people enrolled in the ERAS + narcotic group, and 304 people enrolled in the ERAS group. Using regression analysis, patients enrolled in the narcotics group (IRR = 1.242, p = 0.001) or the ERAS + narcotics group (IRR = 1.886, p < 0.001) were associated with a significantly longer length of stay compared to the ERAS group. Logistic regression analyzed factors associated with 30-day readmission. Negative binomial regression was performed to analyze the length of stay. A grouped Charlson Index score of 1 (IRR = 1.285, p < 0.001) or 2 or higher (IRR = 2.000, p < 0.001) is also associated with a longer length of stay. Other covariates, including age, race, BMI, and smoking status, did not show statistically significant associations in the regression analysis. Patients in the ERAS + narcotics group had significantly increased odds of readmission (OR = 3.507, p < 0.001) compared to the ERAS group. Older age groups, specifically 45-64 years (OR = 0.574, p = 0.001) and 65 years and over (OR = 0.439, p < 0.001), were associated with lower odds of readmission compared to the 18-44 years group. A grouped Charlson Index score of 1 (OR = 1.692, p = 0.019) or 2 or higher (OR = 3.086, p < 0.001) was significantly associated with increased odds of readmission.

Table 1 is a summary of patient characteristics of the four groups of interest: ERAS, narcotics, ERAS + narcotics, and none. Counts and percentages are provided with mean values where applicable. Percentages represent the proportion within each group relative to the total sample size of 3,919.

**Table 1:** Patient characteristics across the four groups. ERAS: Enhanced Recovery After Surgery.

	ERAS	Narcotics	ERAS + narcotics	None	Total
N	304 (7.8%)	1,504 (38.4%)	81 (2.1%)	2,030 (51.8%)	3,919 (100%)
Age group					
18-44	99 (32.6%)	462 (30.7%)	32 (39.5%)	592 (29.2%)	1,185 (30.2%)
45-64	156 (51.3%)	710 (47.2%)	38 (46.9%)	904 (44.5%)	1,808 (46.1%)
65 and over	49 (16.1%)	332 (22.1%)	11 (13.6%)	534 (26.3%)	926 (23.6%)
Sex					
Female	304 (100%)	1,504 (100%)	81 (100%)	2,030 (100%)	3,919 (100%)
Race category					
White	57 (18.8%)	323 (21.5%)	15 (18.5%)	511 (25.2%)	906 (23.1%)
Hispanic	133 (43.8%)	556 (37.0%)	29 (35.8%)	788 (38.8%)	1,506 (38.4%)
Other	114 (37.5%)	625 (41.6%)	37 (45.7%)	731 (36.0%)	1,507 (38.5%)
Payer type					
Medicare/Medicaid	80 (26.3%)	552 (36.7%)	20 (24.7%)	821 (40.4%)	1,473 (37.6%)
Private insurance	124 (40.8%)	482 (32.0%)	23 (28.4%)	596 (29.4%)	1,225 (31.3%)
Other	100 (32.9%)	470 (31.2%)	38 (46.9%)	613 (30.2%)	1,221 (31.2%)
Discharge disposition					
Ex	0 (0.0%)	8 (0.5%)	0 (0.0%)	3 (0.1%)	11 (0.3%)
Home	303 (99.7%)	1,458 (96.9%)	77 (95.1%)	1,990 (98.0%)	3,828 (97.7%)
Hospice	0 (0.0%)	8 (0.5%)	1 (1.2%)	5 (0.2%)	14 (0.4%)
Hospital	1 (0.3%)	30 (2.0%)	3 (3.7%)	32 (1.6%)	66 (1.7%)
Charlson Index, mean (Std)	0.875 (1.54)	1.133 (1.98)	1.198 (2.09)	1.028 (1.72)	1.060 (1.82)
Grouped Charlson Index					
Index = 0	195 (64.1%)	932 (62.0%)	49 (60.5%)	1,203 (59.3%)	2,379 (60.7%)
Index = 1	33 (10.9%)	149 (9.9%)	8 (9.9%)	281 (13.8%)	471 (12.0%)
Index >= 2	76 (25.0%)	423 (28.1%)	24 (29.6%)	546 (26.9%)	1,069 (27.3%)
BMI >= 30					
No	157 (51.6%)	826 (54.9%)	40 (49.4%)	1,141 (56.2%)	2,164 (55.2%)
Yes	147 (48.4%)	678 (45.1%)	41 (50.6%)	889 (43.8%)	1,755 (44.8%)
BMI, mean (Std)	30.75 (6.99)	30.09 (6.67)	30.77 (7.02)	30.12 (6.93)	30.17 (6.83)
Smoking status					
Current	20 (6.6%)	154 (10.2%)	13 (16.0%)	177 (8.7%)	364 (9.3%)
Former	49 (16.1%)	204 (13.6%)	4 (4.9%)	307 (15.1%)	564 (14.4%)
Never	235 (77.3%)	1,146 (76.2%)	64 (79.0%)	1,546 (76.2%)	2,991 (76.3%)

The summary statistics for each patient group are summarized in Table 2 and are categorized by participation in the ERAS protocol, narcotics administration, and the combination of ERAS with narcotics. Percentages represent the proportion of patients within each category relative to the total sample size of 3,919.

**Table 2:** Summary statistics for patient groups. ERAS: Enhanced Recovery After Surgery.

Patient group	Summary
N	3,919
ERAS	
No	3,615 (92.2%)
Yes	304 (7.8%)
Narcotics	
No	2,415 (61.6%)
Yes	1,504 (38.4%)
ERAS + narcotics	
No	3,838 (97.9%)
Yes	81 (2.1%)

Table 3 provides descriptive statistics for low-frequency outcomes, including mortality, DVT, and PE, across patient groups. Outcomes are reported as counts and percentages relative to the total sample size of 3,919 patients. These variables are included for descriptive purposes due to their low occurrence rates and may not be included in regression analyses. Table 4 shows regression outcomes, including the length of stay and 30-day readmission rates, categorized by the patient groups. Table 5 presents the results of a negative binomial regression on length of stay. The analysis indicates that being in the narcotics group (IRR = 1.242, p = 0.001) or the ERAS + narcotics group (IRR = 1.886, p < 0.001) is associated with a significantly longer length of stay compared to the ERAS group. A grouped Charlson Index score of 1 (IRR = 1.285, p < 0.001) or 2 or higher (IRR = 2.000, p < 0.001) is also associated with a longer length of stay.

**Table 3:** Descriptive statistics for low-frequency outcomes, including mortality, deep vein thrombosis (DVT), and pulmonary embolism (PE), across patient groups. ERAS: Enhanced Recovery After Surgery.

	ERAS	Narcotics	ERAS + narcotics	None	Total
N	304 (7.8%)	1,504 (38.4%)	81 (2.1%)	2,030 (51.8%)	3,919 (100%)
Mortality					
No	304 (100%)	1,496 (99.5%)	81 (100%)	2,027 (99.9%)	3,908 (99.7%)
Yes	0 (0.0%)	8 (0.5%)	0 (0.0%)	3 (0.1%)	11 (0.3%)
DVT					
No	298 (98.0%)	1,470 (97.7%)	79 (97.5%)	1,983 (97.7%)	3,830 (97.7%)
Yes	6 (2.0%)	34 (2.3%)	2 (2.5%)	47 (2.3%)	89 (2.3%)
PE					
No	301 (99.0%)	1,484 (98.7%)	77 (95.1%)	2,004 (98.7%)	3,866 (98.6%)
Yes	3 (1.0%)	20 (1.3%)	4 (4.9%)	26 (1.3%)	53 (1.4%)

**Table 4:** Regression outcomes based on the length of stay and 30-day readmission rates. ERAS: Enhanced Recovery After Surgery.

Regression outcomes by patient group					
	ERAS	Narcotics	ERAS + narcotics	None	Total
N	304 (7.8%)	1,504 (38.4%)	81 (2.1%)	2,030 (51.8%)	3,919 (100%)
Length of stay in days, mean (Std)	2.582 (2.852)	3.391 (5.306)	5.210 (5.463)	2.773 (2.945)	3.046 (4.090)
Patient readmitted within 30 days					
No	285 (93.8%)	1,434 (95.3%)	65 (80.2%)	1,910 (94.1%)	3,694 (94.3%)
Yes	19 (6.2%)	70 (4.7%)	16 (19.8%)	120 (5.9%)	225 (5.7%)

**Table 5:** Comparison of the negative binomial (NB) and zero-inflated negative binomial (ZINB) models to evaluate the length of stay (in days) due to overdispersion and the presence of zero-length stays. *** p < 0.01, ** p < 0.05, * p < 0.1. The chi-square statistical test was used for this statistical analysis. ERAS: Enhanced Recovery After Surgery; IRR: incidence rate ratio.

Discharge day	IRR	Robust St. error	z	p-value	95% CI		Sig
Group							
Narcotics	1.242	.079	3.42	.001	1.097	1.406	***
ERAS + narcotics	1.886	.228	5.25	0	1.489	2.391	***
None	1.047	.061	0.79	.431	.934	1.173	
Age							
45-64	.941	.038	-1.49	.136	.869	1.019	
65 and over	1.03	.062	0.50	.62	.915	1.16	
Race							
Hispanic	.994	.049	-0.12	.901	.902	1.095	
Other	1.05	.054	0.94	.345	.949	1.163	
BMI							
BMI greater than or equal to 30	.962	.033	-1.13	.259	.899	1.029	
Smoking status							
Former smoker	.889	.075	-1.39	.164	.754	1.049	
Never smoker	.966	.054	-0.62	.537	.865	1.078	
Grouped Charlson Index score							
Charlson Index = 1	1.285	.058	5.57	0	1.177	1.404	***
Charlson Index >= 2	2	.102	13.56	0	1.81	2.211	***
Constant	2.282	.153	12.30	0	2.001	2.603	***
lnalpha	-1.028	.07	.b	.b	-1.165	-.891	

Table 6 demonstrates the results of a logistic regression analyzing factors associated with 30-day readmission. Factors such as length of surgery, difficulty of surgery, and intraoperative complications were not considered, as the postoperative pain management was comparable (ERAS protocol management vs. ERAS + narcotic management vs. only narcotic management) regardless of these factors. Only surgeries for benign conditions were considered, with comparable groups of laparotomies versus laparoscopies. The results show that being in the ERAS + narcotics group significantly increases the odds of readmission (OR = 3.507, p < 0.001) compared to the ERAS group (readmission is analyzed regardless of specific diagnosis). Older age groups, specifically 45-64 years (OR = 0.574, p = 0.001) and 65 years and over (OR = 0.439, p < 0.001), are associated with lower odds of readmission compared to the 18-44 years group. Older patients may receive more comprehensive care, discharge planning, medications, and follow-ups tailored to their profile, hence returning less compared to the younger group.

**Table 6:** Results of a logistic regression analyzing factors associated with 30-day readmission. *** p < 0.01, ** p < 0.05, * p < 0.1. The chi-square statistical test was used for this statistical analysis. ERAS: Enhanced Recovery After Surgery.

Readmitted	Odds ratio	Robust St. error	z	p-value	95% CI		Sig
Group							
Narcotics	.728	.195	-1.18	.237	.43	1.232	
ERAS + narcotics	3.507	1.261	3.49	.000	1.734	7.094	***
None	.955	.246	-0.18	.859	.577	1.583	
Age group							
45-64	.574	.093	-3.41	.001	.417	.789	***
65 and over	.439	.092	-3.94	.000	.291	.661	***
Race							
Hispanic	1.137	.21	0.69	.488	.791	1.632	
Other	.993	.193	-0.03	.972	.679	1.454	
BMI							
BMI greater than or equal to 30	.945	.135	-0.39	.695	.714	1.251	
Smoking status							
Former	1.031	.325	0.10	.922	.556	1.913	
Never	1.264	.328	0.90	.367	.76	2.101	
Grouped Charlson Index							
Index = 1	1.692	.381	2.34	.019	1.089	2.629	**
Index >= 2	3.086	.522	6.66	.000	2.215	4.298	***
Constant	.052	.019	-8.20	.000	.026	.106	***

A grouped Charlson Index score of 1 (OR = 1.692, p = 0.019) or 2 or higher (OR = 3.086, p < 0.001) is significantly associated with increased odds of readmission. Other variables, such as race, BMI, and smoking status, did not show statistically significant associations in this model.

## Discussion

The ERAS protocol is a multidisciplinary approach to perioperative care to improve surgical outcomes [4]. It is an evidence-based pathway that provides comprehensive care starting from prior to surgery to minimize the stress of surgery, expedite recovery, and decrease complications [2]. The pathways of the ERAS model include preoperative, intraoperative, and postoperative considerations. Preoperative considerations include patient education, optimization, and fasting guidelines. The standardized intraoperative considerations include analgesia, prophylaxis for nausea and vomiting, fluid optimization, thromboprophylaxis (such as using sequential compression devices during and after surgery as well as administering prophylactic Lovenox per Caprini score result), antimicrobial therapy, avoidance of drains and vaginal packs, and maintenance of normothermia. Postoperative considerations include early ambulation, early introduction of regular diet and chewing gum, as well as stepwise, multimodal pain management to minimize opioid administration, fluid optimization, removal of urinary catheter within 24 hours, and defined discharge pathways. The utilization of the ERAS protocol compared to narcotic treatment in the postoperative course of benign gynecological surgeries decreases complications, and therefore is intended to promote decreased use of opioid narcotics.

Excessive narcotic prescription, including medications such as codeine, morphine, oxymorphone, and hydrocodone, for postoperative pain control may contribute to opioid use disorder, defined as the chronic use of opioids that cause distress or impairment [5]. In fact, postoperative opioid dependence or overdose is a significant health problem, with an incidence of about two per 1000 opioid-naive surgical patients prescribed an opioid and followed for five years [6]. Adverse effects related to excessive opioid use in the management of postoperative pain include sedation, respiratory depression, delirium, and ileus [7]. According to Hah et al., preoperative opioid use was associated with longer hospital admissions, a higher rate of readmission, and increased healthcare costs. Persistent opioid use after surgery includes the potential for abuse and addiction [7]. Over-prescription of opioids for acute pain is a primary source of diversion in the United States [8]. Opioid use disorder may escalate to opioid addiction and increase patient morbidity and mortality [9].

Other outcomes, such as mortality, DVT, and PE, were of low frequency. Further research may be done across a larger sample size to determine the effect of using the ERAS protocol on these outcomes. Further research may also investigate the role of narcotics dependence created unintentionally through over-reliance on narcotics for postoperative pain control. With the development of pathways such as the ERAS protocol, dependence on narcotic medication may be decreased, and optimal pain management may be obtained. Readmission rates were also shown to be decreased, which decreases hospital costs. In particular, chewing gum is part of the postoperative management of the ERAS protocol and is currently not available to order within the hospital. We suggest implementing chewing gum as part of an official EMR order to provide to patients as part of their postoperative care.

## Conclusions

To conclude, the utilization of the ERAS protocol compared to narcotic treatment in the postoperative course of benign gynecological surgeries is superior to narcotic treatment and narcotic treatment combined with the ERAS protocol. The ERAS group was associated with shorter length of stay and decreased rates of readmission. The ERAS group was not associated with lower rates of DVT. Implementing the ERAS protocol as a standard of care is an important step shown to decrease hospital costs, improve patient outcomes, and improve hospital quality.

Due to the benefits of the ERAS protocol, it should be implemented widely as a standard of care in our hospital. As this project studied patients undergoing gynecologic surgery, due to the benefits of this approach, we believe the use of the ERAS protocol may be widened to any type of surgery. We believe that this will increase hospital quality and effective care.
